# Histidine Hydrogen-Deuterium Exchange Mass Spectrometry for Probing the Microenvironment of Histidine Residues in Dihydrofolate Reductase

**DOI:** 10.1371/journal.pone.0017055

**Published:** 2011-02-16

**Authors:** Masaru Miyagi, Qun Wan, Md. Faiz Ahmad, Giridharan Gokulrangan, Sara E. Tomechko, Brad Bennett, Chris Dealwis

**Affiliations:** 1 Case Center for Proteomics and Bioinformatics, Case Western Reserve University, Cleveland, Ohio, United States of America; 2 Department of Pharmacology, Case Western Reserve University, Cleveland, Ohio, United States of America; 3 Department of Ophthalmology and Visual Sciences, Case Western Reserve University, Cleveland, Ohio, United States of America; University of Wales Bangor, United Kingdom

## Abstract

**Background:**

Histidine Hydrogen-Deuterium Exchange Mass Spectrometry (His-HDX-MS) determines the HDX rates at the imidazole C_2_-hydrogen of histidine residues. This method provides not only the HDX rates but also the p*K*_a_ values of histidine imidazole rings. His-HDX-MS was used to probe the microenvironment of histidine residues of *E. coli* dihydrofolate reductase (DHFR), an enzyme proposed to undergo multiple conformational changes during catalysis.

**Methodology/Principal Findings:**

Using His-HDX-MS, the p*K*_a_ values and the half-lives (*t*_1/2_) of HDX reactions of five histidine residues of apo*-*DHFR, DHFR in complex with methotrexate (DHFR-MTX), DHFR in complex with MTX and NADPH (DHFR-MTX-NADPH), and DHFR in complex with folate and NADP^+^ (DHFR-folate-NADP^+^) were determined. The results showed that the two parameters (p*K*_a_ and *t*_1/2_) are sensitive to the changes of the microenvironment around the histidine residues. Although four of the five histidine residues are located far from the active site, ligand binding affected their p*K*_a_, *t*_1/2_ or both. This is consistent with previous observations of ligand binding-induced distal conformational changes on DHFR. Most of the observed p*K*_a_ and *t*_1/2_ changes could be rationalized using the X-ray structures of apo-DHFR, DHFR-MTX-NADPH, and DHFR-folate-NADP^+^. The availability of the neutron diffraction structure of DHFR-MTX enabled us to compare the protonation states of histidine imidazole rings.

**Conclusions/Significance:**

Our results demonstrate the usefulness of His-HDX-MS in probing the microenvironments of histidine residues within proteins.

## Introduction

Histidine residues can be useful probes for investigating conformational changes in proteins for two primary reasons. First, the hydrogen-deuterium exchange (HDX) rate of the imidazole C_2_-hydrogen varies as a function of solvent accessibility [1]. Second, the acid dissociation constant (p*K*_a_) of a histidine imidazole group changes in response to the adjacent ionizable group(s) [2], providing an indicator of the local electrostatic environment of the histidine residue. Despite having these unique properties, histidine residues have rarely been used to probe conformational changes in proteins. Presumably, this is because, until the advent of His-HDX-MS, only nuclear magnetic resonance (NMR) spectroscopy [1], [2] was able to determine the HDX rates of imidazole C_2_-hydrogen in proteins.

More than four decades ago, it was found that the p*K*_a_ of the imidazole N-H groups can be determined by measuring the apparent HDX rates at the imidazole C_2_-position at various pH (pD) [3], [4], [5], thus determining both the HDX rate and p*K*_a_ simultaneously. ^1^H NMR spectroscopy has been the analytical technique of choice to monitor the HDX reaction at the imidazole C_2_-position [1]. However, NMR-based methods have difficulties in assigning resonance signals to individual imidazoles and require large amounts and high concentrations of protein.

Our recent proof-of-concept study with a model protein, RNase A, demonstrated that electrospray mass spectrometry can be used to monitor the HDX reactions of histidine imidazoles in proteins [6]. The method has recently been successfully applied to investigate the structural changes in anthrax protective antigen induced by its binding to a anthrax toxin receptor [7] and in rhodopsin upon photon absorption [8]. In the present study, we demonstrate how His-HDX-MS can be used for investigating the changes in the microenvironment of histidine residues of DHFR due to ligand-induced conformational changes.

**Figure 1:**
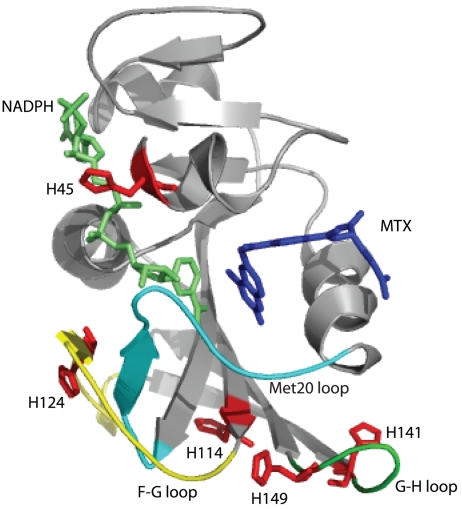
Structure of DHFR-MTX-NADPH complex (PDB: 3DAU). Five histidine residues are shown (red sticks) [31]. MTX and NADPH are shown in blue and lime, respectively. The Met-20 loop (residues 9–24), the F–G loop (residues 116–132) and the G–H loop (residues 142–150) are shown in cyan, yellow and green, respectively.

DHFR catalyzes the reduction of 7,8-dihydrofolate (DHF) through hydride transfer from reduced nicotinamide-adenine dinucleotide phosphate (NADPH) to produce 5,6,7,8-tetrahydrofolate (THF) [9]. DHFR was chosen for our study because conformational changes and long-range concerted motions are associated with catalysis [10], [11]. For example, based on an ensemble of X-ray structures, DHFR is proposed to transition between an open, occluded and closed conformation during catalysis [12]. Furthermore, single molecule fluorescence [13], and X-ray and neutron crystallography [12], [14] have observed an open and closed comformational isoforms of DHFR when bound to the chemotherapeutic drug methotrexate (MTX). The fluctuation between these different states of DHFR is due to conformational changes occurring at a regulatory loop called the Met20 loop (residues 9–24) and other loops such as the F–G (residues 116–132) and G–H loops (residues 142–150) [12], [15], [16]. Regions of these loops are far from the ligand-binding site, further demonstrating how conformational changes are propagated to disparate and distant parts of the DHFR molecule. As DHFR contains five histidine residues that are distributed close to the active site and these loops, they can be used as probes to provide insights into the conformational changes associated with ligand binding (Figure 1). In particular, His45 is located near the active site and has direct contact with the cofactor NADPH/NADP^+^. The other four histidines are located near or within the F–G and G–H loops (Figure 1).

We report here the p*K*_a_ values and half-lives (*t*_1/2_) of HDX reactions of all five histidine residues in apo*-*DHFR, DHFR-MTX, DHFR -MTX-NADPH, and DHFR-folate -NADP^+^. These complexes provide representative snapshots of the open (apo-DHFR) and various closed forms of the enzyme [15], [17]. The present results demonstrate the utility of His-HDX-MS for probing the microenvironment of histidine residues of proteins. This method can be used for studying important biological processes such as signal transduction, receptor-drug interactions, enzyme catalysis, protein folding, and opens the door for histidine scanning for investigating protein structure-function relationships.

## Results and Discussion

### Assignment of HDX rates to the five histidine residues of DHFR

All five histidine residues were detected in four different peptides identified by LC-MS/MS (Figure S1). Initially, the peptides were assigned to the amino acid sequence of DHFR by matching the experimentally obtained peptide masses to their expected molecular masses. The tandem mass spectra for these peptides ensured that the assignments were correct (Figure S2). The four peptides were named His45-, His114-, His124- and His141&149-peptides, based on the positions of five histidines of DHFR. Peptide ions used for calculating the rate of HDX at each of the histidine residues are shown in Table S1.

**Figure 2:**
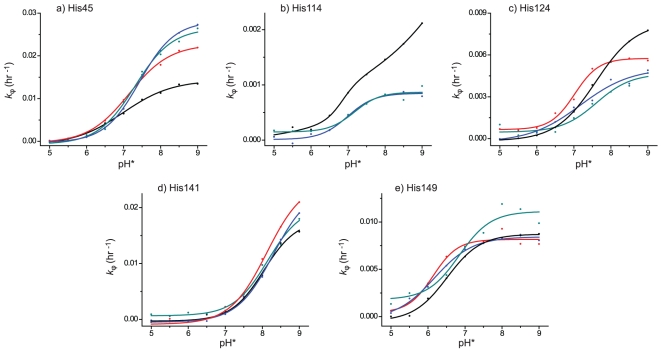
pH* dependence of the *k*_ϕ_ for HDX at the imidazole C_2_-position of five histidine residues in apo-DHFR, DHFR-MTX, DHFR-MTX-NADPH and DHFR-folate-NADP^+^. The His45 (a), 114 (b), 124 (c), 141 (d) and 149 (e) from apo-DHFR (black), DHFR-MTX (red), DHFR-MTX-NADPH (blue), and DHFR-folate-NADP^+^ (green).

The pseudo-first-order rate constants (*k*_ϕ_) for the HDX of the imidazole C_2_-hydrogen of His45, His114 and His124 were calculated directly from the mass spectra of His45-, His114- and His124-peptides. As His141 and His149 occur in the same peptide, the *k*_ϕ_ values for them were derived using tandem mass spectrometry where multiple product ions that contain solely His141 or His149 were produced from the His141&149-peptide (Figure S2d). Among the product ions produced, the b_11_ ion for His141 and y_10_ ion for His149 were chosen to obtain the *k*_ϕ_ values, because the intensities of these two ions were among the highest observed. Thus, tandem mass spectrometry was used to provide the *k*_ϕ_ values at individual histidine residues in peptides containing multiple histidine residues. The *k*_ϕ_ values are plotted as a function of pH* (direct pH meter readings of the D_2_O buffer solutions calibrated with standard buffer solutions made with H_2_O) for each histidine residue in Figure 2. All of the histidine residues gave simple sigmoid curves corresponding to a single p*K*_a_ except for His114 in apo-DHFR (Figure 2b). The enhanced *k*_ϕ_ values above pH 8 for His114 may indicate greater solvent accessibility, which could be attributed to possible local conformational change at alkaline pH. Furthermore, we could not obtain an interpretable sigmoid curve for His114 in DHFR-MTX due to the extremely slow HDX rate. This is because based on the solvent accessible surface area His114 is more buried in the DHFR-MTX complex compared to the apo-DHFR and the other DHFR complexes.

### Histidine p*K*_a_ values

Measured p*K*_a_ values from the sigmoid curves of individual histidine residues are shown in Table 1. Interestingly, there are several ligand induced p*K*_a_ changes to the histidine residues that were observed. First, the p*K*_a_ of His45 increased at least 0.31 pH units upon MTX-NADPH or folate-NADP^+^ binding, whereas the increment was less significant (0.16 pH unit) upon MTX binding. Second, the p*K*_a_ of His124 decreased at least 0.43 pH units upon MTX and MTX-NADPH binding, but not upon folate-NADP^+^ binding. Third, the p*K*_a_ of His149 decreased at least 0.34 pH units upon MTX and MTX-NADPH binding, while the p*K*_a_ changed 0.33 pH units upward upon folate-NADP^+^ binding. Note that we did not convert the pH* to pD to calculate the p*K*_a_s, since the p*K*_a_ values calculated with pH* are fairly close to those determined in H_2_O [18], [19]. This is because the constant term of 0.4 in pH*/pD conversion (pD = pH*+0.4) [20] is approximately canceled by a decrease of acidities of acids in D_2_O [18].

**Table 1:** p*K*_a_ values of histidine residues in apo-DHFR, DHFR-MTX, DHFR-MTX-NADPH and DHFR-folate-NADP^+^ complex.

	*pK_a_*			
	DHFR	DHFR-MTX	DHFR-MTX-NADPH	DHFR-folate-NADP^+^
His45	6.99 (±0.07)	7.15 (±0.04)	7.43 (±0.03)	7.30 (±0.07)
His114	6.84 (±0.20)	N.D.	6.99 (±0.10)	7.17 (±0.16)
His124	7.60 (±0.03)	7.02 (±0.06)	7.17 (±0.20)	7.61 (±0.19)
His141	8.04 (±0.08)	8.12 (±0.14)	8.29 (±0.10)	8.14 (±0.11)
His149	6.51 (±0.04)	6.13 (±0.13)	6.17 (±0.10)	6.84 (±0.17)

### Relationship between p*K*_a_ and microenvironment

#### Histidine 45

**Figure 3:**
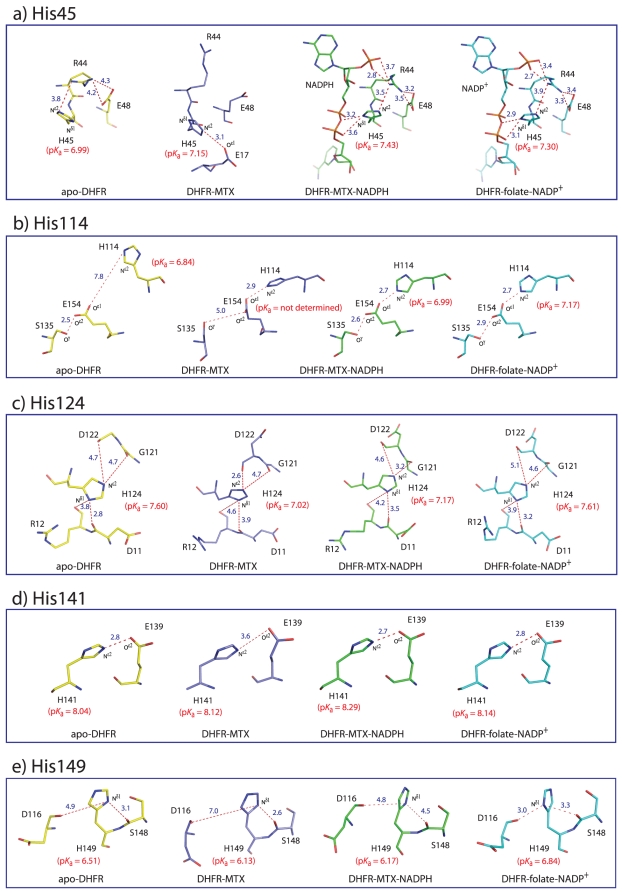
Microenvironment of histidine residues in apo-DHFR, DHFR-MTX, DHFR-MTX-NADPH and DHFR-folate-NADP^+^ structures. a) His45, b) His114, c) His124, d) His141, and e) His149. The carbon atoms of apo-DHFR, DHFR-MTX, DHFR-MTX-NADPH and DHFR-folate-NADP^+^ are shown in yellow, lightblue, green, and cyan, respectively.

Figure 3a shows the microenvironment of His45 in the crystal structures of apo-DHFR, DHFR-MTX, DHFR-MTX-NADPH, and DHFR-folate-NADP^+^, respectively. The imidazole ring of His45 in the DHFR-MTX-NADPH and DHFR-folate-NADP^+^ structures is located close to the negatively charged pyrophosphoryl moiety of NADPH/NADP^+^; the distances between N^δ1^ of His45 and the two oxygen atoms of the pyrophosphoryl moiety are within 3.5 Å. It has been well established that neighboring groups that increase the electron density of the imidazole ring (e.g., RCOO^−^, RO^−^, RS^−^) increase the p*K*_a_ of the imidazole ring and that neighboring groups that decrease the electron density of the imidazole ring (e.g., RNH_3_^+^, GdnH^+^, ImH^+^) decrease the p*K*_a_ of the imidazole ring [21]. Thus, the increased p*K*_a_ of His45 in the NADPH/NADP^+^ bound DHFRs are possibly due to the close proximity of the imidazole ring of His45 with the pyrophosphoryl moiety of NADPH/NADP^+^. The p*K*_a_ of this histidine residue in DHFR-MTX is lower than those in DHFR-MTX-NADPH and DHFR-folate-NADP^+^, but still 0.16 pH units higher than that in apo-DHFR even though there are no NADPH/NADP^+^ molecules. The close distance from the carboxyl group of Glu17 to His45 (3.1 Å) may be the reason for the higher p*K*_a_ in DHFR-MTX than expected. This glutamic acid residue (Glu17) is more than 13 Å away in DHFR-MTX-NADPH and DHFR-folate-NADP^+^, therefore it is unlikely to affect the p*K*_a_ of His45. Unfortunately, Glu17 is not observed in the apo-DHFR structure, possibly due to the enhanced flexibility of the Met20 loop, destabilized by the absence of ligand binding. We predict a decreased p*K*_a_ for His45 in apo-DHFR relative to binary and ternary complexes because the Met20 loop is undergoing rapid conformational switching between open and closed forms (∼200 s^−1^). Thus, there is no ability to form any strong binding contacts.

#### Histidine 114

With the exception of the DHFR-MTX complex, the p*K*_a_ values of His114 were almost the same in apo-DHFR and the other complexes, and the values were slightly higher than the intrinsic p*K*_a_ value of a fully solvent exposed histidine residue (p*K*_a_≈6.5) [6]. As discussed previously, the p*K*_a_ of His114 was unable to be measured in the DHFR-MTX complex due to slow exchange rates as the imidazole side chain is more buried relative to apo-DHFR and the other complexes. There is no electron-donating or electron-withdrawing group found near His114 in apo-DHFR. The closest one is the side-chain carboxyl group of Glu154 that is 7.8 Å away (Figure 3b). Whereas in other three structures, the O^ε1^ of Glu154 is localized close proximity of the N^ε2^ of His114 (within 3 Å). Despite its proximity to Glu154 the p*K*_a_ values of this histidine residue are not elevated large extent as is seen on His141 (see below). We hypothesize that a hydrogen bonding network exists among His114, Glu154 and Ser135, in which O^γ^ of Ser135 donates a hydrogen to O^ε2^ of Glu154 and then O^ε1^ of Glu154 donates a hydrogen to N^ε1^ of His114 to form the hydrogen bonds; consequently, there is no electron-donating effect of the carboxyl group of Glu154 to His114.

#### Histidine 124

The p*K*_a_ values of His124 in DHFR-MTX and DHFR-MTX-NADPH were lower than apo-DHFR, while the p*K*_a_ remained the same when folate-NADP^+^ was bound. His124 is surrounded by the backbone carbonyl oxygen atoms of Asp11, Arg12, Gly121 and Asp122 in all the four structures (Figure 3c). These are the only electron-donating atoms around His124 and no electron-withdrawing groups were found. Based on the crystal structures we are unable to explain why the p*K*_a_ of His124 in DHFR-MTX and DHFR-MTX-NADPH are lower than those in apo-DHFR and DHFR-folate-NADP^+^.

#### Histidine 141

The p*K*_a_ of His141 in all the DHFR samples were high (≥8) compared to the intrinsic p*K*_a_ value of a fully solvent exposed histidine residue (p*K*_a_≈6.5) [6], indicating there is a negatively charged group or groups in close proximity to this histidine. As expected, in all structures the imidazole ring of His141 is in close proximity to Glu139 (Figure 3d), suggesting that it forms a salt-bridge with this presumably charged glutamic acid residue at neutral pH.

#### Histidine 149

The p*K*_a_ of His149 in DHFR-MTX and DHFR-MTX-NADPH were significantly lower than in apo-DHFR, while the p*K*_a_ increased upon folate-NADP^+^ binding. As the electron density on the imidazole ring is affected by surrounding electronegative atoms, their distances to the N^δ1^ atom may correlate with p*K*_a_. In fact, the combined distances to the carbonyl oxygen atoms of Asp116 and Ser148 from the N^δ1^ of His149 appear to correlate well with the observed p*K*_a_ of His149 (Figure 3e). The order of distances are as follows: DHFR-MTX (9.6 Å)>DHFR-MTX-NADPH (9.3 Å)>apo-DHFR (8.0 Å)>DHFR-folate-NADP^+^ (6.3 Å). The longer combined distances correlate well with lower p*K*_a_ values. These results suggest that the electron-donating effects by the backbone carbonyl oxygens of Asp116 and Ser148 to His149 is the determinant factor of the p*K*_a_ of the side chain of His149.

### Histidine *t*_1/2_ values

The *t*_1/2_ values for the 5 histidine residues of apo-DHFR, and the DHFR binary and ternary complexes are shown in Table 2. The significant changes in *t*_1/2_ due to ligand binding are: 1) the *t*_1/2_ of His45 decreased greater than 1.7-fold upon MTX, MTX-NADPH and folate-NADP^+^ binding, 2) the *t*_1/2_ of His114 increased at least 2-fold upon MTX, MTX-NADPH and folate-NADP^+^ binding, and 3) the *t*_1/2_ of His124 increased at least 1.4-fold upon MTX, MTX-NADPH and folate-NADP^+^ binding.

**Table 2:** Half-lives of H-D exchange at histidine residues in apo-DHFR, DHFR-MTX, DHFR-MTX-NADPH and DHFR-folate-NADP+ complex.

	*t_1/2_ (day)*			
	apo-DHFR	DHFR-MTX	DHFR-MTX-NADPH	DHFR-folate-NADP^+^
His45	2.21 (±0.07)	1.27 (±0.05)	1.02 (±0.01)	1.09 (±0.04)
His114	16.57*	>50	34.07 (±1.64)	33.22 (±2.31)
His124	3.46 (±0.06)	5.01 (±0.14)	5.71 (±0.61)	6.20 (±0.67)
His141	1.62 (±0.10)	1.17 (±0.12)	1.22 (±0.09)	1.42 (±0.12)
His149	3.31 (±0.05)	3.54 (±0.13)	3.42 (±0.08)	2.60 (±0.17)

#### Histidine 45

The *t*_1/2_ values of His45 in DHFR-MTX, DHFR-MTX-NADPH and DHFR-folate-NADP^+^ were lower than apo-DHFR, suggesting that His45 has greater solvent accessibility (ability of bulk solvent to access the particular histidine imidazole ring) in the binary and tertiary complexes than in apo-DHFR. His45 is located close to the negatively charged pyrophosphoryl moiety of NADP^+^/NADPH in the crystal structures of DHFR-MTX-NADPH and DHFR-folate-NADP^+^ (Figure 3a). The negatively charged pyrophosphoryl moiety is expected to stabilize the cationic imidazolium of His45. Since the rate of the HDX reaction at the imidazole C_2_-position is directly proportional to the concentration of cationic imidazolium [4], the close proximity of the pyrophosphoryl moiety of NADPH/NADP^+^ to His45 is likely to be the contributing factor for the higher exchange rates of the ligand bound ternary complexes compared to apo-DHFR. The higher exchange rate of His45 in DHFR-MTX compared to apo-DHFR may be its close proximity to the carboxyl group of Glu17. However, since this glutamic acid residue cannot be seen in apo-DHFR structure, no conclusion can be made.

#### Histidine 114 and 124

**Figure 4:**
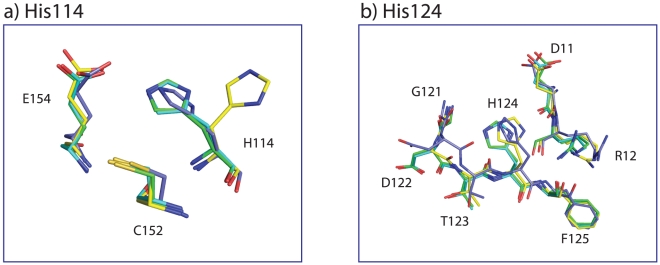
Ligand-induced conformational changes of His114 (a) and 124 (b). The carbon atoms of apo-DHFR, DHFR-MTX, DHFR-MTX-NADPH and DHFR-folate-NADP^+^ are shown in yellow, lightblue, green, and cyan, respectively.

In contrast to His45, the *t*_1/2_ of His114 and His124 increased upon MTX, MTX-NADPH and folate-NADP^+^ binding, suggesting that the solvent accessibilities of these histidine residues decreased. Examination of the crystal structures of apo-DHFR, DHFR-MTX, DHFR-MTX-NADPH, and DHFR-folate-NADP^+^ demonstrated that upon ligand binding the side-chain of His114 underwent a conformational change (Figure 4a), that resulted in the formation of a new hydrogen-bond between the imidazole N^ε2^ atom and the O^ε1^ of Glu154 and a contact with the side chain methylene group of Cys152. These interactions appear to contribute to the slower HDX in the ligand bound structures. In apo-DHFR, His114's imidazole side-chain faces the solvent. A similar trend was noticed for His124, where upon ligand binding there is a conformational change of the imidazole side-chain leading to contacts with residues 121-123 (Figure 4b). It should be noted that solvent permeation and local fluctuation events are assumed to be important determinants of HDX of proteins [22], the contribution of which cannot always be predicted from the structural data.

#### Histidine 141 and 149

The *t*_1/2_ of His141 and 149 were almost the same in apo-DHFR and the other complexes. The side chain of His141 is exposed to the bulk solvent and no notable differences are observed in its microenvironment among the four crystal structures (Figure 3d), thus consistent with our observations on its comparable p*K*_a_ and *t*_1/2_ values in the apo-DHFR and the other complexes. On the other hand, as discussed previously, we observed subtle differences in the electrostatic environment around His149 between structures (Figure 3e) that were sufficient to cause the changes in its p*K*_a_ but not its *t*_1/2_ values. Thus, the results show that the subtle differences in the electrostatic environment did not alter the solvent accessibilities of His149.

### Comparison of His-HDX-MS with NMR and neutron crystallography data

We are able to compare our findings for the DHFR-MTX complex with NMR and neutron crystallography studies. Poe and co-workers determined the p*K*_a_ of five histidine residues in *E. coli* DHFR complex with MTX using ^1^H NMR [23]. The assignments of the five histidine C_2_-NMR resonances were done based on the local electrostatic environments of the five histidine residues in the crystal structure of DHFR-MTX [24]. The p*K*_a_ values assigned to the five histidine residues are not consistent with those determined by His-HDX-MS. This might be because the p*K*_a_ assignments in the NMR study were made based on the electrostatic environment of the five histidine residues derived from the DHFR-MTX crystal structure. Since the assignments of p*K*_a_ values to the individual histidine residues by His-HDX-MS is straightforward and no assumptions are involved [6], we are confident that the p*K*_a_ assignments in our experiments are correct. Other than *E. coli* DHFR, numerous NMR studies have been performed on *Lactobacillus casei* DHFR including a study determining the p*K*_a_ of seven histidine residues in this enzyme [25]. Unfortunately we are unable to do a suitable comparison with this work due to considerable sequence variability between the two species.

**Figure 5:**
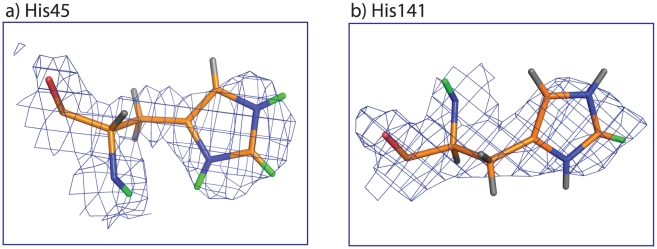
Nuclear density map of His45 and 141 in DHFR-MTX taken from PDB (ID: 2INQ). Deuterium and hydrogen atoms are shown as green and gray sticks, respectively. Other atoms are carbon (orange), nitogen (blue) and oxygen (red).

Since neutron diffraction provides an experimental method of directly locating deuterium atoms in proteins including the C_2_-deuteron of histidine [26], we analyzed the neutron diffraction data of DHFR-MTX complex [14]. As we expected, from the nuclear density maps, it was evident that the C_2_-position of His45 and 141, which were the two residues that showed the fastest HDX (Table 2), undergo HDX (Figure 5a and b). The other residues did not show significant nuclear densities at the C_2_-positions, which correlates well with the slow-exchanging histidines in our study. Based on the His-HDX-MS experiments conducted at 37°C over 3 days, His114 hardly undergoes HDX, while His45, 124, 141 and His149 approximately undergo 80, 34, 83, 44% (calculated from the *k*_ϕ_^max^ values of these residues), respectively. The crystals in the neutron crystallography study underwent HDX at 4°C for 4 weeks. Since the rate of HDX at 4°C is predicted to be much slower than the rate at 37°C, the extent of deuteration in the crystal is expected to be lower than that at 37°C for 3 days. Hence, it is not surprising that poor nuclear density was observed for His114, His124 and His149.

### Global conformational changes upon ligand binding

It has been proposed that long-range molecular dynamics can provide energy for enzymatic reactions. DHFR has been proposed to be such an enzyme [9], [27]. Studies such as X-ray crystallography [12], NMR relaxation experiments [17] and amide HDX mass spectrometry [28], have indicated that the conformation of DHFR changes continuously throughout the course of the enzymatic reaction. As mentioned before, the major sites of conformational change include the active site loop (residues 9-24, termed the Met-20 loop), the F-G loop (residues 116-132) and the G-H loop (residues 142-150). These loop regions are distant from the active site. Except for His45, which has direct contact with the cofactor, the other histidine residues are at least 10 Å away from the active site (see Figure 1). His124 is in the F-G loop and His114 is at the base of the F-G loop. His149 is in the G-H loop and His141 is at the base of the G-H loop. We observed dramatic effects on either p*K*_a_ or *t*_1/2_ or both on the five histidine residues upon ligand binding. Our results, therefore, indicate that even though the Met20 loop in all the ligand-bound complexes analyzed here adapt a closed conformation, considerable differences in their electrostatic environment and/or solvent accessibilities exists.

We have also compared the HDX rates at C_2_-position of the five histidine residues with the amide HDX rates on the peptides that contain those histidine residues [29]. No correlation was found between the results. This is not surprising because His-HDX rates are specific to the side chain of histidine residues, whereas amide HDX rates are summed HDX rates on all the backbone amides in the peptides that contain one or more of those histidine residues.

### His-HDX-MS for structural biology

Our results demonstrate that with only sub-nanomole amounts of sample, His-HDX-MS can provide unambiguous assignments of p*K*_a_ and *t*_1/2_ for individual histidine residues in proteins, and the two parameters (p*K*_a_ and *t*_1/2_) are extremely sensitive to changes in the microenvironment around histidine residues. Therefore, this method should be useful to study the changes in the microenvironment of histidine residues due to conformational changes induced by ligand binding and protein-protein interactions. We postulate that this method is applicable to proteins that are not amenable to analysis by NMR spectroscopy such as large proteins/protein complexes (MW>100 kDa), complex protein mixtures like whole cell lysates, and proteins that are available in limited amount. An obvious limitation of His-HDX-MS is that the technique relies on the presence of histidine residues at sites or near the sites of interest. Many histidine residues may be surface exposed and “uninteresting” (i.e., show no change in their p*K*_a_ and *t*_1/2_ values upon ligand binding), or simply the frequency of histidine residues in proteins of interest may be too low for the technique to describe global structural changes. We think the limitation of the technique can be overcome by introducing histidine residues at the sites of interest using site-directed mutagenesis. Such a ‘His-scanning’ approach will be tested in the future. Another limitation is the long incubation time required for sufficient HDX (at least several hours [6]). This means that His-HDX-MS cannot detect dynamical changes that occur on a short time-scale. This is an inherent limitation of the method. Moreover, the requirement of long incubation times will be problematic for proteins that are not stable during the incubation. Further improvement of mass spectrometry instrumentation that allows us to measure smaller changes in the M and M+1 ratio of peptide peaks will be needed in order to shorten the required incubation time.

## Materials and Methods

### Material

Deuterium oxide (D_2_O, 99.9%) was purchased from Cambridge Isotope Laboratories (Andover, MA), and deuterium chloride (DCl) and sodium deuteroxide (NaOD) were purchased from Sigma-Aldrich (St. Louis, MO). Folate and methotrexate (MTX) were purchased from Sigma-Aldrich. Immobilized chymotrypsin was purchased from Princeton Separations (Adelphia, NJ) and *Staphylococcus aureus* V-8 protease (Glu-C) was purchased from Thermo Fisher Scientific (Waltham, MA). All other chemicals and materials used were either reagent grade or were of the highest quality that was commercially available.

### Preparation of *E. coli* DHFR and DHFR-ligand complexes

*E. coli* DHFR was expressed and purified using a similar protocol for expression and purification of *B. anthracis* DHFR, as we described previously in [30]. Briefly, the cDNA was cloned into the Champion pET-Sumo vector (Invitrogen, Carlsbad, CA) and then transformed into chemically competent BL21(DE3) *E. coli* cells (Novagen-EMD Biosciences, Gibbstown, NJ) for IPTG-inducible protein expression. The overexpressed protein was purified using an immobilized Nickel affinity column (Ni-NTA; Qiagen, Valencia, CA), and the 6xHis-SUMO tag attached to the N-terminus of DHFR was cleaved by ULP1 protease in the presence of a surfactant, IGEPAL CA-630 (Sigma-Aldrich). The cleaved DHFR protein was separated from the non-cleaved DHFR by a subsequent pass over immobilized Nickel affinity resin, and the IGEPAL was removed from the protein by weak anion exchange chromatography (DEAE2; Bio-Rad, Hercules, CA). As a polishing step, concentrated protein was injected onto a Superdex-75 gel filtration column (GE Healthcare, Piscataway, NJ) and the middle peak fractions were pooled for the HDX-MS experiments. DHFR-MTX, DHFR-MTX-NADPH and DHFR-folate-NADP^+^ complexes were prepared as follows. Due to the poor solubilities of MTX and folate in aqueous solvent, a five-fold molar excess of these ligands were added as a solid to the apoenzyme while the DHFR concentration was relatively dilute, 0.75–1.5 mg/mL. After a short incubation with the ligands, the DHFR-ligand complexes were then concentrated 10-fold with a YM10 Centricon (Millipore, MA, USA) device. The cofactors (NADP^+^ or NADPH at a five-fold molar excess) were added directly to the concentrated protein.

### Buffer solutions in D_2_O

Buffers, 50 mM MES (pH 5.0–7.5) and 50 mM HEPES (pH 8.0 - 9.0), were made with 99.9% D_2_O. All buffers contained 50 mM NaCl. The pH of the buffers was adjusted with diluted DCl or NaOD and measured with a Solution Analyzer Model 4603 (Amber Science, Eugene, Oregon) equipped with a glass/AgCl electrode (Model 476086, Nova Analytics, Woburn, MA). The final D_2_O content of the buffers were assumed to be 99.9%, neglecting the exchangeable hydrogens attached to heteroatoms of MES and HEPES that remained in the buffers. The reported pH* values are direct pH meter readings of the D_2_O buffer solutions calibrated with standard buffer solutions made with H_2_O and are uncorrected for the isotope effect at the glass electrode.

### Assessment of stability of DHFR and its complexes by circular dichroism spectroscopy

We have studied the stability of Apo-DHFR, DHFR-MTX, DHFR-MTX-NADPH and DHFR-folate-NADP^+^. The proteins were incubated at 37°C for 72 hrs. Then, the CD spectra were monitored at the far-UV wavelengths. Far-UV CD spectra of apo DHFR and its complexes display a minimum between 215–220 nm, indicating β-sheet structure. A representation of the CD spectra at pH 7.0 is shown in Supporting Information Figure S3, which demonstrates that DHFR retains its predominantly β-structure over the 72 hour time course.

### Deuteration and digestion of DHFR

Apo-DHFR, DHFR-MTX, DHFR-MTX-NADPH and DHFR-folate-NADP^+^ (650 pmol in H_2_O) were diluted 10-times with D_2_O, of which 4 µL (260 pmol) were mixed with 36 µL of buffer with different pH* values (pH 5.0–9.0), and incubated at 37°C for 72 hr. The final D_2_O content in the reaction mixture was approximately 99%, assuming no HDX through moisture in the atmosphere. The duration of incubation was set to be sufficiently long for determining the HDX rate constant (*k*_ϕ_) of His114, which is the slowest exchanging histidine residue in DHFR. We have demonstrated that the HDX reaction follows pseudo-first-order kinetics, and a linear relationship between the HDX rate and the incubation time can be obtained with a model peptide [6]. The reaction was stopped by mixing with 5 µL formic acid, and the protein was freed from the buffer salts using an Ultra Micro Spin C18 column (Nest Group, Southboro, MA) according to the manufacturer's instructions and dried in a Speed Vac. The protein was redissolved in 20 µL 0.1 M ammonium bicarbonate and digested with 0.25 µg immobilized chymotrypsin at 25°C for 1 hr. After the digestion the solution was centrifuged at 3,000× *g* for 1 min in a tabletop centrifuge and the supernatant was recovered. The recovered chymotryptic peptides were further digested by 1 µg of V8 protease for another 1 hr at 25°C. The resulting digest solution was dried in a Speed Vac and redissolved in 800 µL 0.1% TFA and analyzed by LC-MS/MS as described below.

### LC-MS/MS

The digests prepared above were analyzed by LC-MS/MS using a UltiMate 3000 LC systems (Dionex, San Francisco, CA) interfaced to a LTQ-Orbitrap XL mass spectrometer (Thermo-Finnigan, Breman, Germany). The platform was operated in the nano-LC mode using the standard nano-ESI API stack fitted with a picotip emitter (uncoated fitting, 10 µm spray orifice, New Objective Inc., Woburn, MA). The solvent flow rate through the column was maintained at 300 nL/min using a 1∶1000 splitter system. The protein digests (5 µL) were injected into a reversed-phase C18 PepMap trapping column (0.3×5 mm, 5 µm particle size, Dionex) equilibrated with 0.1% TFA/2% acetonitrile (v/v) and washed for 5 min with the equilibration solvent at a flow rate of 25 µL/min, using an isocratic loading pump operated through an autosampler. Note that the use of 0.1% TFA instead of 0.1% formic acid was required to retain one of the histidine containing peptides (His45 peptide) onto the trapping column. After the washing step, the trapping column was switched in-line with a reversed-phase C18 Acclaim PepMap 100 column (0.075×150 mm, Dionex) and the peptides were chromatographed using a linear gradient of acetonitrile from 2% to 50% in aqueous 0.1% formic acid over a period of 40 min at 300 nL/min and the eluate was directly introduced into the mass spectrometer. The mass spectrometer was operated in a data-dependent MS to MS/MS switching mode, with the two most intense ions in each MS scan subjected to MS/MS analysis. The full MS scan was performed at 60000 resolution and the subsequent MS/MS analysis was performed at 30000 resolution. The total scan cycle frequency was approximately 1 sec. The precursor ion isolation width was set to be m/z±2.0 that allowed to transmit the M and M+2 isotopic ions of the peptide for CID. The threshold intensity for the MS/MS trigger was set at 2000 and the fragmentation was carried out using the CID mode using a normalized collision energy (NCE) of 35. The data was entirely collected in the profile mode. Dynamic exclusion function for previously selected precursor ions was not enabled during the analysis. Xcalibur software (version 2.0.5, build 0704, Thermo-Finnigan) was used for instrument control, data acquisition, and data processing.

### Measurement of p*K*_a_ and half-life (*t*_1/2_) of HDX reaction

The pseudo-first-order rate constant (*k*_ϕ_) of HDX reaction was determined by monitoring the changes in the ratios of M+1/M isotopic peak of a given peptide before (time = 0) and after (time = *t*) the HDX reaction. The intensities of the M and M+1 peaks at time *t* = 0 are represented as *I*_M_(0) and *I*_M+1_(0), respectively, and the intensities of the same peaks at time *t* are represented as *I*_M_(*t*) and *I*_M+1_(*t*). Since the HDX reaction follows pseudo-first-order kinetics, the intensities of *I*_M_(*t*) and *I*_M+1_(*t*) can be expressed by equation 1 and 2, respectively.
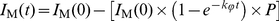
(1)

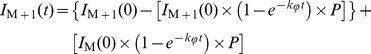
(2)where *P* is the fractional D_2_O content in the solvent (P = 1 when D_2_O content is 100%). By taking the ratio *I*_M+1_(*t*)/*I*_M_(*t*) and arranging the equation for *k*_ϕ_, the equation 3 is derived.
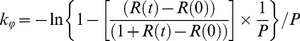
(3)where *R*(0) = *I*_M+1_(0)/*I*_M_(0) and *R*(*t*) = *I*_M+1_(*t*)/*I*_M_(*t*), and *t* is the incubation time (hr). Note that only a single histidine residue is allowed in the peptide to determine *k*_ϕ_ at the peptide level. The equation is fundamentally the same as the equation 4 in our previous report [6], but the term *P* (fractional D_2_O content) is incorporated into the equation to correct the *k*_ϕ_ value upward when *P*<1.

The p*K_a_* value was obtained from the sigmoid titration curve plotted *k*_ϕ_ versus pH* using Origin Graphing software (Ver. 8.0, OriginLab, Northampton, MA) using the following equation.
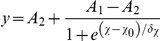
where A_1_ is the minimum rate constant at the lowest pH, A_2_ is the maximum rate constant at the highest pH, *x*_0_ is the point of inflection, and δ*_x_* is the change in *x* corresponding to the most significant change in *y* values.

The half-life (*t*_1/2_, day) of the exchange reaction was estimated using the equation: *t*_1/2_ (day) = ln2/*k*_ϕ_^max^/24, where *k*_ϕ_^max^ (hr^−1^) is the maximum rate constant obtained from the plateau to the alkaline side of the sigmoid titration curve.

### Structural Analysis

The comparison of protein structures was performed using the program PyMOL (Molecular Graphics System software, DeLano Scientific, Palo Alto, CA). The structural data of apo-DHFR (PDB code number: 5DFR), DHFR-MTX (PDB code number: 2INQ), DHFR-MTX-NADPH (PDB code number: 3DAU) and DHFR-folate-NADP+ (PDB code number: 1RX2) as deposited in the Protein Data Bank were used in the comparison.

## Supporting Information

Figure S1 **Representative LC-MS/MS total ion current chromatogram of peptides derived from DHFR.** The protein digest (3.1 pmol) was injected into a reversed-phase C18 PepMap trapping column (0.3×5 mm, 5 µm particle size, Dionex) equilibrated with 0.1% TFA/2% acetonitrile (v/v) and washed for 5 min with the equilibration solvent at a flow rate of 25 µL/min. After the washing step, the trapping column was switched in-line with a reversed-phase C18 Acclaim PepMap 100 column (0.075×150 mm, Dionex) and the peptides were chromatographed using a linear gradient of acetonitrile from 2% to 50% in aqueous 0.1% formic acid over a period of 40 min at 300 nL/min and the eluate was directly introduced into the mass spectrometer. The full MS scan was performed at 60000 resolution and the subsequent MS/MS analysis was performed at 30000 resolution. The precursor ion isolation width was set to be m/z±2.0 that allowed to transmit the M and M+2 isotopic ions of the peptide for CID.
(EPS)

Figure S2 **Tandem mass spectra of His45- (a), His114- (b), His124- (c) and His141&149-peptide (d).** Precursor ions were m/z 328.7 (*z* = 2), 513.8 (*z* = 2), 1028.9 (*z* = 2) and 804.3 (*z* = 2) for His45-, His114-, His124- and His141&149-peptide, respectively. Only b and y series product ions are labeled.
(EPS)

Figure S3 **Circular dichroism (CD) spectra of apo-DHFR (A), DHFR-MTX (B), DHFR-MTX-NADPH (C), and DHFR-folate-NADP^+^ (D).** The spectra were measured after a 72 hr incubation period in D_2_O buffer at 37°C. Black line represents CD spectra at 0 hr time point whereas red line represents CD spectra after incubation at 37°C for 72 hr.
(EPS)

Table S1 **Histidine-containing peptides/peptide fragments monitored.** *His141&149-peptide (Ser138 - Tyr151) contains His141 and 149. The amino acid sequences, positions and monitored ions shown for His141 and 149 are for the fragment ions (b11 and y10, respectively) produced from the peptide by collision-induced dissociation.
(XLS)
